# Impact of a Statewide Internet-Based Tobacco Cessation Intervention

**DOI:** 10.2196/jmir.9.4.e28

**Published:** 2007-09-30

**Authors:** Jessie E Saul, Barbara A Schillo, Sharrilyn Evered, Michael G Luxenberg, Annette Kavanaugh, Nathan Cobb, Lawrence C An

**Affiliations:** ^5^University of Minnesota Medical SchoolMinneapolisMNUSA; ^4^Pulmonary and Critical Care UnitMassachusetts General HospitalBostonMAUSA; ^3^Professional Data AnalystsIncMinneapolisMNUSA; ^2^Center for PreventionBlue Cross and Blue Shield of MinnesotaSaint PaulMNUSA; ^1^ClearWay MinnesotaMinneapolisMNUSA

**Keywords:** Tobacco use cessation, Internet, behavior, evaluation studies

## Abstract

**Background:**

An increasing number of people have access to the Internet, and more people are seeking tobacco cessation resources online every year. Despite the proliferation of various online interventions and their evident acceptance and reach, little research has addressed their impact in the real world. Typically, low response rates to Internet-based follow-up surveys generate unrepresentative samples and large confidence intervals when reporting results.

**Objectives:**

The aim of this study was to achieve a high response rate on follow-up evaluation in order to better determine the impact of an Internet-based tobacco cessation intervention provided to tobacco users in Minnesota, United States.

**Methods:**

Participants included 607 men and women aged 18 and over residing in Minnesota who self-reported current tobacco use when registering for an Internet-based tobacco cessation program between February 2 and April 13, 2004. Participants were given access to an interactive website with features including social support, expert systems, proactive email, chat sessions, and online counselors. Mixed-mode follow-up (online survey with telephone survey for online nonrespondents) occurred 6 months after registration.

**Results:**

Of the study participants, 77.6% (471/607) responded to the 6-month follow-up survey (39.4% online and 38.2% by telephone). Among respondents, 17.0% (80/471, 95% CI = 13.6%-20.4%) reported that they had not smoked in the past 7 days (observed rate). Assuming all nonrespondents were still smoking (missing=smoking rate), the quit rate was 13.2% (80/607, 95% CI = 10.5%-15.9%).

**Conclusions:**

This mixed-mode follow-up survey of an online smoking cessation program achieved a high response rate and provides a more accurate estimate of long-term cessation rates than has been previously reported. Quit rates for the Internet-based tobacco cessation program were higher than those expected for unassisted quit attempts and are comparable to other evidence-based behavioral interventions. The similarities between quit rates demonstrates that an Internet-based cessation program may have as great an impact as, and can have wider reach than, other cessation programs such as those delivered by telephone. With over 100000 people having visited the website and over 23000 having registered, a 6-month self-reported quit rate of 13.2% suggests that the quitplan.com program helped over 3000 Minnesotans remain tobacco free for at least 6 months. Results of this study suggest that an Internet-based cessation program is a useful tool in states’ efforts to provide comprehensive cessation tools for smokers.

## Introduction

An estimated 45.1 million Americans (20.9%) are current smokers [1]. More than 70% of US smokers want to quit, and 4 in 10 try to quit each year [1,2]. Unfortunately, most individuals who attempt to quit do so without receiving evidence-based treatments such as telephone quitlines, in-person counseling options, and pharmaceutical products [3]. Success rates for these unassisted quit attempts are low [4-7].

The Internet is a promising channel for improving delivery of tobacco treatment services. Approximately 60% of American adults reported having Internet access in the home in 2004 [8], and nearly 70% of US adults reported using the Internet at least occasionally in 2005 [9]. Searching for health information online is common [8], and it is estimated that as of 2004 over 8 million people had searched online for help to stop smoking [10].

The population impact of tobacco control programs is a product of both reach and effectiveness among participants [11]. Despite the proliferation of various online interventions and their evident acceptance and reach [12-16], little research has addressed their impact in the real world. Four recent randomized clinical trials have shown that individually tailored self-help materials delivered over the Internet result in modest increases in short-term abstinence [13,17-19], but information on longer term follow-up is limited. A number of demonstration and pilot projects of online cessation programs have been reported in the literature, with cessation rates ranging from 3% to 18% at time points ranging from 1 to 3 months [12,17,20,21]. However, results from these studies are difficult to interpret because of low response rates (10% to 56%) at follow-up [12,13,16-18,20,22].

While participant attrition is a usual, and even expected, aspect of online health-related applications [23], it poses a unique challenge for studies of online tobacco cessation interventions due to the strong association between response to follow-up and smoking status (more nonresponders are using tobacco than responders) [24-31]. Only one randomized clinical trial has produced response rates greater than 60% at 6 months: in this study, Muñoz et al [32] showed that tailored email messages increased the effectiveness of an online quit smoking guide. However, the study’s use of monetary incentives to promote return to the site as well as the self-selected nature of the participants make the results less generalizable to the larger population of Internet users seeking help online to quit smoking.

ClearWay Minnesota, a nonprofit organization created as part of Minnesota’s legal settlement with the tobacco industry, offers a range of statewide cessation services including Internet services through the quitplan.com website. Since the Internet service was launched in July 2003, over 100000 individuals have visited the site, and over 23000 individuals have registered for the service, making it the most popular of ClearWay Minnesota’s offerings [33]. At the time ClearWay Minnesota began providing Internet cessation services, there was limited information on the effectiveness of these programs. In response, ClearWay Minnesota contracted with an external evaluation firm to conduct an independent evaluation study of quitplan.com. The goal of this study was to determine a more precise estimate of the program’s impact on its participants. This study was designed specifically to address gaps in the current literature by achieving a high response rate at a commonly used follow-up point (ie, 6 months after registration).

## Methods

### Quitplan.com Services

Content and programming for quitplan.com are provided by Healthways QuitNet Inc. The QuitNet service has been described elsewhere [16]. ClearWay Minnesota provides access to premium QuitNet services to all Minnesotans through a branded quitplan.com website. These services include online social support, expert systems, tailoring, and proactive email to enhance both cessation and relapse prevention. In addition, online counselors answer individual questions, and website staff moderate the forums and host chat sessions. Individuals in the quitplan.com program participate in the global social support community of all QuitNet powered websites.

### Recruitment

All registrants included in the study (1) resided in Minnesota, (2) were at least 18 years old, (3) were accessing the site as a current tobacco user, and (4) did not report having already quit at the time of registration. All those reporting being in action or maintenance stages of tobacco cessation were excluded from the study. Of the 1294 registrants during the study period, 288 were not residents of Minnesota, were under 18 years old, were accessing the site on behalf of someone else, or self-identified as an evaluator or researcher. All 1006 eligible registrants who accessed the quitplan.com website between February 2 and April 13, 2004 were shown an additional screen during the registration process inviting them to participate in the study.

**Figure 1:**
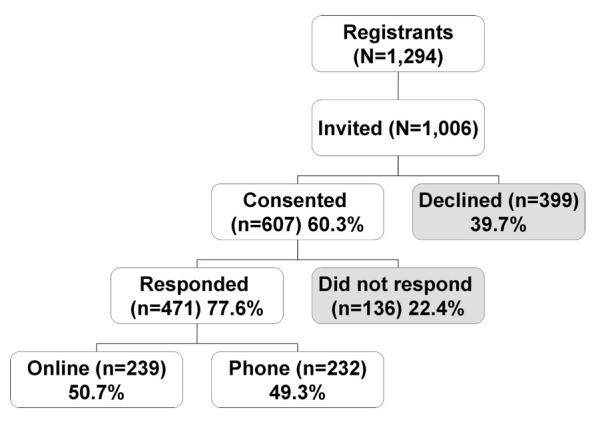
Consent and response rates for study participants with registration dates between February 2 and April 13, 2004 (shaded boxes represent registrants for whom a full data set was not collected due to either lack of consent or response)

Figure 1 illustrates the flow of quitplan.com registrants through the consent and response protocol. A cohort of 607 individuals (60.3%) consented to participate in the study. The rate of consent for studies is often not well documented or reported in the literature. Of those who do report consent rates [13,20,32,34], the rates range between 20.9% and 76.8%. The consent rate for the current study is within the reported range.

### Evaluation

The study design consisted of a mixed-mode follow-up survey using email and, for those not responding by email, telephone. Participants were mailed a pre-notification letter 6 months after program registration and were then sent an email inviting them to complete an online evaluation survey. Reminder emails were sent to nonrespondents 3 and 7 days after this initial email, and attempts were made 12 days after the initial email to contact nonrespondents by phone to complete the follow-up evaluation. Up to 20 attempts to contact nonrespondents were made over multiple days at different times of day. All respondents received a US $10 check for completing the follow-up survey.

### Measures

Three data sources were used for this study: registration data, site usage data, and survey results. Demographic and clinical variables that were collected online at registration included age, gender, education, geographic region, employment status, health insurance status, marital status, readiness to quit (all types of tobacco), cigarettes per day, time to first morning cigarette, frequency of cigarette use, quit history (all types of tobacco), and quit attempts in the past year (all types of tobacco). In addition, this study examined the number of return visits to the site (log-ins) in the 6 months following registration, since the number of log-ins has been shown to be predictive of cessation outcomes in prior analyses [16]. Finally, the follow-up survey assessed quit status and other behaviors (7- and 30-day point prevalence, use of medications or other quit aids since registration, number of quit attempts since registration, and duration of longest quit since registration) 6 months after study enrollment.

The primary outcome measure was self-reported 7-day point prevalence abstinence at 6 months post-registration. Quit status was assessed by self-report, which is consistent with the recommendation of the Society for Research on Nicotine and Tobacco Subcommittee on Biochemical Verification for low-demand studies [35]. Primary analysis of abstinence rates is by assuming all those lost to follow-up are still smoking (missing=smoking). Missing=smoking calculations are standard in the evaluation of cessation programs. We included all those who consented to participate in the study as the denominator for calculating the missing=smoking quit rate. Respondent-only (observed) quit rates are presented for comparison purposes.

### Statistical Analysis

Analysis was performed using SPSS (Statistical Package for the Social Sciences) version 13.0 (SPSS Inc, Chicago, IL, USA). The analysis to calculate quit status and determine which variables were associated with quit status used both forced and forward step-wise logistic regressions. The impact of possible response bias on calculations of quit status was addressed by using a missing=smoking analysis that assumed that individuals not reached for follow-up were still smoking.

Registration and site usage data were examined to assess the degree that response bias influenced cessation outcomes. Baseline characteristics for respondents to the follow-up survey were compared to nonrespondents using chi-square tests for categorical variables and *t* tests or nonparametric tests for continuous variables, as appropriate.

Logistic regression was used to identify predictors of cessation. Several independent variables predictive of quit success in previous research include age [36], gender [37-39], education level [40,41], employment status [42,43], health insurance status [44,45], level of addiction [36,46], stage of readiness to quit [47], level of tobacco use [48,49], quit history [50,51], use of medications [4], and number of log-ins to Internet-based cessation programs [16]. Given the demonstrated correlations, a forced logistic regression analysis was conducted including these variables of interest. The forced model entered the variables in two blocks: all baseline variables (demographic and clinical) in the first block, and self-reported medication use (nicotine replacement therapy [NRT] or prescription medications such as Zyban) and the number of log-ins after registration (tricategorized) in the second block. The intent of this design was to test if log-ins and/or medication use was a significant predictor of quit status after adjusting for known baseline characteristics. Because the majority of registrants never logged in again after registration, the number of log-ins was divided into three categories (never logged in again after registration, 1-3 log-ins after registration, and 4 or more log-ins after registration) to provide groupings that both made sense cognitively and resulted in large enough numbers in each category to be able to conduct analysis. Forward step-wise logistic regression modeling was also performed and yielded essentially the same results. Therefore, the results of the a priori (ie, forced) model are presented here.

### Institutional Review

This study was reviewed by the University of Minnesota’s Institutional Review Board and determined to be exempt under federal guidelines 45 CFR 46.101 (b) for existing data.

## Results

### Response Rates and Response Bias

At 6 months, 471 of the 607 individuals in the study completed a follow-up survey, resulting in a final response rate of 77.6% (39.4% online, 38.2% by telephone). Of those who did not respond online, over half (56.3%) were reached in 3 attempts, and nearly four fifths (79.0%) were reached in 6 attempts. Had the protocol included only 3 attempts, the total response rate would have been 70.7% (429/607). Sample size calculations indicated that a sample of 400 respondents was sufficient to determine 6-month quit rates with a 95% confidence level with a ±5% margin of error.

### Participant Characteristics

Characteristics of respondents and nonrespondents are presented in Table 1. Some items were only assessed at follow-up (eg, marital status, medication use since registration). Of the 471 respondents, most were 25-44 years old (57.3%, 270/471), female (66.0%, 311/471), and lived in the 7-county Minneapolis/St. Paul metro area (63.6%, 288/453). A large majority had some college or postgraduate education (82.8%, 358/432) and were employed for wages (74.9%, 349/466). About half were married (50.4%, 237/470). Nearly all reported primary use of cigarettes (98.5%, 464/471), and most used tobacco daily (80.0%, 373/466). Roughly one quarter of respondents reported either light (27.6%, 130/471) or heavy (21.7%, 102/471) use of cigarettes, while about half reported moderate use (50.7%, 239/471).

Respondents were more likely to be older than nonrespondents (mean age 38.57 vs 35.75 years; *P* = .008), more likely to be insured (88.1% vs 80.9%; *P* = .03), and more likely to have ever quit for 30 days or more at registration (59.2% vs 47.1%; *P* = .01). There were no significant differences between respondents and nonrespondents in gender, geographic location, education level, employment status, type of tobacco used, daily versus occasional smoker, smoking intensity, time to first morning cigarette, stage of readiness to quit, 30-day quit in the past year, or ever quit for a year or more.

Nonresponders differed significantly from responders in terms of their utilization of the website. Nonresponders were more likely to have never logged in again after registration (68.4%, 93/136,) as compared to responders (47.1%, 222/471; *χ* ^2^_2_ = 19.09, *P* < .001).

**Table 1:** Quitplan.com 6-month follow-up survey participant characteristics

Variable	Respondents (n = 471)		Nonrespondents (n = 136)		*P* value ^*^ (respondents vs nonrespondents)	Participants (N = 607)	
	No.	%	No.	%		No.	%

**Age group**					.05		
18-24 years	55	12	27	20		82	14
25-44 years	270	57.3	78	57		348	57.3
45-64 years	139	29.5	30	22		169	27.8
65 or older	7	2	1	1		8	1
**Gender**					.12		
Female	311	66.0	80	59		391	64.4
**Metro vs outstate**					.81		
Outstate	165	36.4	47	38		212	36.7
7-county metro area	288	63.6	78	62		366	63.3
**Education level (trichotomous)**					.58		
High school or less	74	17	26	21		100	18.0
Some college	208	48.1	60	48		268	48.1
College graduate/postgraduate	150	34.7	39	31		189	33.9
**Employed for wages**					.95		
Yes (employed)	349	74.9	100	74.6		449	74.8
**Health insurance status**					.03		
Uninsured	54	12	25	19		79	14
**Married (y/n)^†^**					NA		
Yes (married)	237	50.4	NA			NA	
**Primary form of tobacco used**					.65^‡^		
Cigarettes	464	98.5	135	99.3		599	98.7
Cigars	4	1	0	0		4	1
Pipe	1	0	0	0		1	0
Chewing tobacco or snuff	2	0	1	1		3	1
**Cigarette use: daily or less than daily**					.18		
Daily	373	80.0	115	85.2		488	81.2
**Smoking intensity at registration**					.09		
Light smoker (< 15 cigarettes/day)	130	27.6	38	28		168	27.7
Moderate smoker (15-24 cigarettes/day)	239	50.7	57	42		296	48.8
Heavy smoker (25+ cigarettes/day)	102	21.7	41	30		143	23.6
**Time to first cigarette of the day (at registration )**					.73		
Within 5 min	138	29.3	42	31		180	29.7
6-30 min	198	42.0	61	45		259	42.7
31-60 min	79	17	21	15		100	16.5
After 60 min	56	12	12	9		68	11
**Stage of readiness to quit, 3 categories (at registration)**					.75		
Precontemplation and contemplation	236	50.1	66	49		302	49.8
Preparation	235	49.9	70	52		305	50.2
**Attempted to quit in previous year (from registration)**					.87		
Yes	298	63.3	85	63		383	63.1
**Quit for 30 days or more in past 12 months?**					.52		
Yes	43	9	10	7		53	9
**Ever quit for 30 days or more?**					.01		
Yes	279	59.2	64	47		343	56.5
**Ever quit 1 year or more?**					.38		
Yes	77	16	18	13		95	16
**Number of log-ins in past 6 months (categorical)**					< .001		
Never logged in	222	47.1	93	68		315	51.9
1-3 log-ins	149	31.6	28	21		177	29.2
4 or more log-ins	100	21.2	15	11		115	18.9
**Cessation medication pattern (as reported at 6-month follow-up)^†^**					NA		
None reported	243	51.6	NA			NA	
NRT only	138	29.3	NA			NA	
Zyban only	56	12	NA			NA	
NRT and Zyban	34	7	NA			NA	

NA, not available.

^*^P-values are from *χ*^2^ statistics.

^†^Only asked at follow-up.

^‡^This calculation was done on a very small number of cases with a highly skewed distribution leading to small marginal expected values.

### Cessation Outcomes

Both respondent-only and missing=smoking quit rates are presented here. Among respondents, 17.0% (80/471, 95% CI = 13.6%-20.4%) reported that they had not smoked in the past 7 days at the time of the 6-month follow-up. Using a missing=smoking analysis, the quit rate is 13.2% (80/607, 95% CI = 10.5%-15.9%).

There were no differences between telephone and online respondents in terms of any of the three cessation outcomes (7-day point prevalence, 30-day point prevalence, or 30-day abstinence at some point during the past 6 months).

Results from the logistic regression model predicting 7-day abstinence at 6 months are shown in Table 2. The only variable with a significant odds ratio for 7-day abstinence at follow-up was “number of log-ins after registration.” The odds of having quit were 2.90 (95% CI = 1.45-5.77) times higher for those logging in four or more times after registration at 6 months than for those who never logged in again after registration.

**Table 2:** Odds ratios for forced logistic regression model for 7-day abstinence at 6 months (N = 417)*

	*P* value	Odds Ratio (95.0% CI)
**Number of log-ins**		
1-3 log-ins in past 6 months vs none	.38	1.35 (0.69-2.67)
4+ log-ins in past 6 months vs none	.002	2.90 (1.45-5.77)
**Age**		
Age at registration	.29	0.99 (0.96-1.01)
**Gender**		
Gender (female vs male)	.91	1.04 (0.57-1.89)
**Education**		
Some college vs high school or less	.82	1.10 (0.49-2.47)
College graduate/postgraduate vs high school or less	.90	0.94 (0.39-2.27)
**Employment**		
Employed for pay	.84	1.08 (0.54-2.14)
**Health insurance status**		
Insured	.15	2.29 (0.75-7.02)
**Time to first cigarette of the day**		
6-30 min vs within 5 min	.63	1.19 (0.60-2.37)
31-60 min vs within 5 min	.27	0.58 (0.22-1.54)
After 60 min vs within 5 min	.48	1.43 (0.53-3.90)
**Stage of change**		
Preparation vs contemplation/precontemplation	.96	1.01 (0.57-1.80)
**Smoking intensity**		
Moderate smoker (15-24 cigs/day) vs light smoker (1-14 cigs/day)	.51	0.79 (0.39-1.58)
Heavy smoker (25+ cigs/day) vs light smoker (1-14 cigs/day)	.35	0.64 (0.25-1.64)
**Quit history**		
Ever quit for 30 days or more	.47	1.25 (0.69-2.26)
**Use of medications**		
Used meds (NRT and/or Zyban) in past 6 months	.10	1.63 (0.91-2.95)

^*^11.5% excluded due to missing; Nagelkerke *R* ^2^ = 0.102.

## Discussion

### Principal Results

Several studies suggest that tobacco cessation programs can be delivered effectively via the Internet [13,18,19,32]. However, with the exception of the study by Muñoz et al [32], these studies were limited by large differences between observed and missing=smoking quit rates. For example, Cobb et al (2005) achieved a response rate of only 25.6% at 3 months, resulting in an observed quit rate of 30% and a missing=smoking rate of 7% [16]. The current study achieved a markedly higher response rate (78%), substantially closing the gap between observed (17.0%) and missing=smoking rates (13.2%).

By using a mixed-mode methodology for follow-up at 6 months, the present study resulted in a higher response rate, thus increasing our confidence in the precision of the estimated quit rate. Similar to a recent study by Couper et al (2007), we found that many of those lost to online follow-up can be “brought back” through alternate modes of data collection [52]. It should be noted that our study, while not designed to test for mode effects, found no differences between telephone and online respondents in terms of any of the three cessation outcomes. Couper et al, however, found evidence that use of the telephone produced more socially desirable responses on weight loss outcomes when compared to mail as an alternate mode to online follow-up, pointing to the need to carefully consider mode effects in any future studies of online tobacco cessation interventions.

For the present study, we conclude that the best estimate of 7-day abstinence at 6 months after registration is between 13% (assuming missing=smoking) and 17% (among respondents only, ie, observed). Some have suggested that missing=smoking is an overly conservative approach for follow-up surveys because not all individuals who fail to answer the surveys continue to smoke [53,54]. This is particularly the case when the goal is to evaluate the effectiveness of real-world programs and not to compare different groups in a trial setting. The results of missing=smoking analyses may be considered the lower estimate of program impact just as the observed quit results based on respondents only may be considered an upper estimate.

In the present study, the missing=smoking estimate is comparable to data from evaluations of other cessation programs funded by ClearWay Minnesota. For example, the missing=smoking quit rate for ClearWay Minnesota’s QUITPLAN Helpline (prior to the introduction of free NRT to under- and uninsured callers) was 11.0% [55]. The similarities between quit rates suggest that an Internet-based cessation program may have a greater impact than behavioral-based telephone quitlines that do not provide NRT, given their greater reach (ie, easy access, availability, and participation) and noting that both types of programs produce comparable quit rates.

Several clinical trials are in progress to more fully evaluate the effectiveness of Internet-based cessation programs. Data from these trials will help to identify which elements of Internet-based tobacco cessation programs are critical for enhancing quit success. It may be that certain features or content may reduce effectiveness of the program, as has been shown in other studies [32].

In the present study, the number of log-ins was significantly correlated to quit status. It is interesting to note that an independently verifiable behavior occurring after registration, as opposed to baseline demographics or tobacco history, was predictive of quit status. Additional research is needed to determine the existence and direction of a causal relationship between log-ins and quitting and whether mechanisms for getting people to return to an Internet cessation website might increase the efficacy of the intervention.

### Limitations

As an observational study, participants were not randomized into a control group, which limits the conclusions that can be drawn regarding effectiveness of the quitplan.com website. Muñoz et al have already shown that Internet-based cessation programs are effective [32]. However, this study addresses one limitation of the Muñoz study in that it measures quit rates for Internet users outside of the context of a randomized clinical trial and can thus be more easily generalized to users of Internet-based cessation programs in the real world.

Individuals who agreed to participate in a program evaluation did differ in terms of demographic or smoking-related characteristics and outcomes from those who did not agree to participate. As a result, participants and nonparticipants may be expected to differ in terms of their cessation outcomes. Given that only 60% of those who were invited to participate consented to do so, it may be that all site users have a different rate of abstinence than the subset of those who consented to participate. Future studies should consider strategies to increase initial consent rates to further improve generalizability.

### Conclusions

This mixed-mode survey produced a high response rate, resulting in more accurate estimates of long-term cessation rates than previously reported. Quit rates for the Internet-based tobacco cessation program were better than those expected for unassisted quit attempts and are comparable to other evidence-based interventions. With over 100000 people having visited the site and over 23000 having registered since inception of the program in 2003, a 6-month self-reported quit rate of 13.2% suggests that the quitplan.com program has helped over 3000 Minnesotans remain tobacco free for at least 6 months. Results of this study suggest that an Internet-based cessation program is a useful tool in states’ efforts to provide comprehensive cessation programs for smokers.

## Abbreviations

NRT: nicotine replacement therapy
